# The impact of the 2021 flood on the outpatient care in the North Rhine region, Germany: a cross-sectional study

**DOI:** 10.1186/s12889-023-17279-y

**Published:** 2024-01-22

**Authors:** Luca Theresa Wiesehahn, Andrea Kaifie

**Affiliations:** Institute for Occupational, Social, and Environmental Medicine, Medical Faculty, RWTH Aachen University, Aachen, Germany

**Keywords:** Heavy precipitation, Natural disaster, Health care system, Public health, Questionnaire

## Abstract

**Background:**

In the summer of 2021, heavy precipitation led to extreme flooding across Western Europe. In Germany, North Rhine-Westphalia and Rhineland-Palatinate were particularly affected. More than 180 people lost their lives, and over 700 were left injured and traumatized. In the North Rhine district alone, more than 120 practices were only able to operate to a limited extent or had to close their practices completely. The aim of this study was to assess the impact of the 2021 flood on the outpatient care in the North Rhine region.

**Methods:**

The cross-sectional study was conducted in January 2022 in practices affected by the flood in the North Rhine region. For this purpose, 210 affected practices were identified using a list of the Association of Statutory Health Insurance Physicians for North Rhine (KVNO) and via telephone and/or e-mail. These practices were forwarded a questionnaire that addressed, among other things, the local accessibility of the practices before and after the flood, possibilities of patient care in different premises and received support.

**Results:**

A total of 103 practices (49.1%) returned the completed questionnaire, of which 1/4 were general practitioners. 97% of the practices reported power failure, more than 50% water supply damage and nearly 40% the loss of patient records. 76% of the participating practices stated that they needed to close their practice at least temporarily. 30 doctors took up patient care in alternative premises. The average number of patients seen by doctors per week before the flood was 206.5. In the first week working in alternative premises, doctors saw an average of 66.2 patients (-50.3%). Especially elderly/geriatric patients and patients with disabilities were identified as particularly vulnerable in terms of access to health care after the flood.

**Conclusions:**

The flood had a significant negative impact on outpatient care. We determined not only a high number of closed practices and a large decrease in patient numbers but also differences in patient care assessment depending on the type of alternative premises. To address outpatient care disruptions after extreme weather events more effectively, appropriate measures should be implemented pre-emptively.

## Background

In July 2021, record-breaking heavy precipitation over a period of three days combined with already saturated soils led to severe flooding in Western Europe. In Germany, the federal states North Rhine-Westphalia (NRW) and Rhineland-Palatinate were particularly affected, when several bodies of water overflowed their banks [1, 2]. Based on the hydrological data, the flood event was classified as extreme according to the flood risk management of the Ministry of the Environment, Nature Conservation and Transport of North Rhine-Westphalia [3]. By definition, a natural disaster of this dimension is expected to occur less than once in 100 years. Compared to past flood events in Germany, the recent flooding caused one of the highest casualties counts and was among the worst natural disasters the country has experienced in decades [4]. During the Oder flood of 1997, which caused economic damage of 320 million euros in Germany, more than 100 people in neighboring countries lost their lives [5, 6]. In 2002 the Elbe-flood caused the deaths of more than 20 people in Saxony [7]. In contrast, the flood in July 2021 cost the life of over 180 people in Germany alone. Furthermore, over 800 people were seriously injured and even more may have been traumatized [4, 8, 9]. Several localities were destroyed, the infrastructure was severely affected, and many inhabitants had to live in damaged houses partly without gas, electricity and water supply or were even left without a home at all [10, 11]. After the water receded and as cleanup began, it became clear that the storm had left behind several hundred thousand tons of bulky waste [12–14] and an insurance loss of 8.5 billion euros [15, 16].

In addition to the destruction in other areas, there was also a great deal of impairment in the health care system, which at the time already had to cope with the corona pandemic. The rising water, power outages triggered by this and failing emergency power generators endangered patients, especially those being ventilated in intensive care units [10, 17–19]. Therefore, some hospitals in NRW were forced to evacuate a part or all of their patients. Evacuation was among other things complicated by the lack of digital patient records, forcing the receiving hospitals to rely on little, partly handwritten information. Subsequently, some of the hospitals had to remain closed and were unable to or limited in providing patient care for the duration of the renovations.

Yet not only inpatient medical care was affected by the flood, but also outpatient doctors, psychologists and psychotherapists [10, 20–22]. The incoming water destroyed complete practices, their medical equipment, private patient records and drugs such as vaccines. Many practices were without gas, electricity and water supply and part of the doctors were additionally affected with their private households. Occupied with cleanup activities or due to a lack of facilities, many doctors were unable to care for patients as usual, sometimes for extended periods of time. According to the Association of Statutory Health Insurance Physicians for North Rhine (KVNO), as a result of the flood *„[…] [a]round 120 practices in North Rhine were unable to operate at all or could only operate to a limited extent, and more than 40 of them were completely destroyed […]”* ([20], in the following, all German quotations are translated to English by the authors). Nonetheless, the KVNO announced a few days after the disaster that *“[…] outpatient medical care–and in particular general care and outpatient surgical care – [was] ensured […] “* [23].

Concerning outpatient care, the German health care system is organized as follows. Outpatient care is provided by practice-based specialists and doctors. Some specialists are also affiliated with a local hospital, for example to provide surgery. All of them are members of one of Germanys 17 regional Associations of Statutory Health Insurance Physicians (KVs), as the KVNO [24, 25]. These are institutions of medical self-administration that are responsible for ensuring the accessibility, quality and economic viability of outpatient care. Apart from monitoring the quality of doctors' services, the KVs also represent the interests of the doctors towards the Health Insurance companies, distribute the doctors’ payments and organize on-call duties.

So far, only little information exists on the effects of flooding or other extreme weather events regarding the outpatient care. The aim of this study was to assess the impact of the flood in 2021 on outpatient care in the North Rhine region.

## Methods

This cross-sectional study was conducted in January 2022 approximately six months after the flood. The Ethics Committee of the Medical Faculty of the Rheinisch-Westfälische Technische Hochschule Aachen (RWTH University) approved the study, and the voluntary nature of participation was pointed out to all survey participants.

### Participant recruitment

In the period after the flood, doctors’ offices and psychotherapists reported flood-related damage of their practices to the KVNO. As a result, a list of affected medical practices could be compiled to inform their patients [26]. This list with status as of 30.09.2021 was used as a guide in order to identify the areas in the district North Rhine most affected by the flood in the field of outpatient care. On Google Maps, we located further neighboring practices close to water bodies that overflowed their banks or in regions regarded as high-risk areas for flooding based on calculated risk maps, partly provided by the city councils [3, 27].

The doctors and psychotherapists from the list of the KVNO as well as neighboring practices with strong indicators of flood damage on their websites were included. The remaining practices located in the affected regions were contacted via phone or e-mail. Inclusion criteria were as follows: reported water in the rooms, closure of the practice due to the flood or circumstances resulting from the flood that they by themselves defined as limitations for patient care (such as local accessibility, staff shortage, telephone and power failure, noise disturbance, etc.). Subsequently, we included previously unidentified practices that were located in the neighborhood of affected practices and identifiable as affected when the questionnaires were distributed in person.

Practices were excluded if the contact person assured that flood-related restrictions did not affect practice operations or a clear expression of unwillingness to participate in the survey was communicated. A total of 210 practices were identified as flood affected. To increase the response rate, we delivered 116 (55.2%) paper-based questionnaires together with a self-addressed and stamped return envelope to the affected practices in Stolberg, Eschweiler, Euskirchen, Erftstadt and Leverkusen in person. The remaining 44.8% (94) of the included practices received the questionnaires by mail. Prior to delivery, practices were notified by e-mail or fax. There was no compensation for the participation in the survey.

### Questionnaire

Due to the lack of comparable research and the short time interval to the event, the development of the questionnaire was based on media and news reviews of occurred damages and limitations. The aim was to identify barriers that could have prevented the maintenance of quality outpatient care after the flood. In addition to the resulting disruptions to practice operations, we were interested in the implementation of announced support. The resulting 37-item questionnaire included four main sections:

(A) **Information on the participating practices and the situation before the flood;** including specialization, practice type, number of patients and home visits per week, local accessibility as well as the outpatient care in the region in general surveyed on the basis of practice and pharmacy density, waiting times for elective appointments and accessibility by phone or e-mail;
(B) **Consequences of the flood**; including disruptions and material damages as well as the closure of the practice and the replacement situation,
(C) **Outpatients care in the period after the flood; **including the use of alternative premises, possibilities of patient care, patient numbers, workload and the safeguarding of outpatient care in the region,
(D) **Measures and financial support to secure outpatient care; **focusing on what was known, what was applied for and what was received or used.

Questions concerning specialization, type of practice, type of damage, factors influencing the closing of the practice, type of alternative premises, and vulnerable patient groups were queried via a list selection. It was indicated at the end of the question whether a multiple choice or a single answer was desired. In addition, a significant share of the data was surveyed via agreement to certain statements. These statements refer to the local accessibility of the practice before and after the flood or of the alternative premises instead, the possibilities of patient care in above-mentioned premises, and the situation in the region before and after the flood. The inquiry was conducted using a four-point Likert scale including ‘completely untrue’, ‘rather untrue’, ‘rather true’, and ‘completely true’. Data on the overall assessment of outpatient care assurance after the flood was collected via the German school grading system, which ranges from 1 = very good to 6 = unsatisfactory. Moreover, open questions were used to capture, for example, the number of patients, house visits or the closing time. Participants also had the opportunity to give free comments on certain questions and the topic of outpatient care after the flood in general.

### Data cleansing and preparation

Of the 210 questionnaires distributed we received 103 completed questionnaires back (response rate: 49.1%). The data was transferred into a data matrix and subjected to a process of data cleansing and preparation. *N* = 6 questionnaires were excluded since they did not meet the inclusion criteria. The question about the main field of work in terms of specialization generated a certain number of dual answers. Particularly common was the combination of general medicine and internal medicine, probably due to the fact that in addition to general practitioners, internists can also work as family doctors. Hence, we have interpreted the additional response of 'general medicine' as 'working as a family doctor' and included all practices that had indicated both specializations in the category 'general medicine', aiming to best represent the actual distribution. Regarding the type of alternative premises, two questionnaires with a dual selection were also considered. Concerning the assessment of the possibilities of patient care depending on the type of premises, these were both assigned to the ‘no practice’-type. Altogether, data from 97 questionnaires was included in this analysis.

### Statistical analyses

SAS Software (SAS® OnDemand for Academics; SAS Institute Inc., USA) was used to collect and analyze the data. We performed descriptive analyses of all items to quantify the characteristics of the participating practices and the experiences made in the period after the flood. Associations between variables (such as amount of damage and duration of closure as well as type of alternative premises and possibilities of patient care) or subgroups (as example, those who had to close or not and those who used alternative premises or not) were analyzed. One-way analysis of variance (ANOVA) with the test of homogeneity of variance according to Levene and the Welch’s test as well as the Tukey’s range test was used to investigate statistically significant differences. All statistical tests were based on a significance level of *p* < 0.05.

## Results

The 97 participating practices whose data was included in the analysis comprised predominantly general practitioners (24.7%, *n* = 24), gynecologists (13.4%, *n* = 13), internists (12.4%, *n* = 12) and psychiatrists / psychotherapists (10.3%, *n* = 10) (Table 1). 55.7% (*n* = 54) of the doctors stated to have worked in individual practices, 29.9% (*n* = 29) in group practices and 6.2% (*n* = 6) in medical care centers.

**Table 1:** Group characteristics regarding practice specializations and type

**Group characteristics**	**All participating practices** (*n* = 97, *n*_*a*_ (%))
**Specialization**	
General medicine	24 (24.7)
Psychiatry and psychotherapy	10 (10.3)
Gynecology and obstetrics	13 (13.4)
Internal medicine	12 (12.4)
Ophthalmology	7 (7.2)
ENT (Ear, Nose and Throat)	4 (4.1)
Neurology	4 (4.1)
Pediatrics	4 (4.1)
Child and adolescent psychiatry and psychotherapy	4 (4.1)
Surgery incl. OMS (oral and maxillofacial surgery)	4 (4.1)
Orthopedics	3 (3.1)
Radiology	3 (3.1)
Urology	3 (3.1)
Dermatology	2 (2.1)
**Practice type**	
Individual practice	54 (55.7)
Group practice	29 (29.9)
Others ^a^	14 (14.5)

^a^ (medical care center, interregional group practice, etc.)

**Fig. 1:**
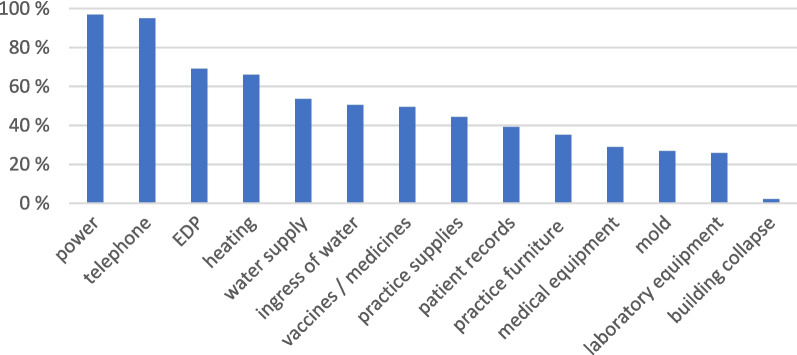
Absolute frequency of disruptions and damages reported by the practices, from most to least frequent

We considered 14 possible damages and disruptions resulting from the flood and asked the participating practices, via list selection, which of these they had experienced. Most frequently, the practices indicated that they had been affected by power failure (96.9%, *n* = 94), telephone failure (94.9%, *n* = 92) and electronic data processing (EDP) failure (69.1%, *n* = 67). The remaining damages and disruptions that were listed in our questionnaire are displayed in Fig. 1.

On average, participating practices were affected by 6.8 of the 14 queried disruptions and damages while 26 doctors (26.8%) reported being affected by 10 or more. Additional disruptions in the participants’ private households were indicated by 33.7% (*n* = 32). From these 32 responders, 84.4% (*n* = 27) had to close their practice temporarily or permanently. *n* = 10 stated (38.5%) that disruptions in their private household were one of the reasons for closure.

**Fig. 2:**
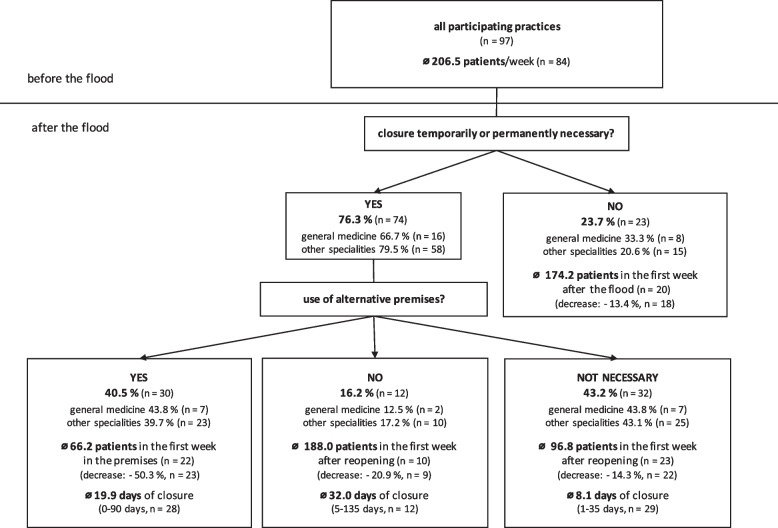
Number of patients before and after the flood and closure time in different subgroups. Indicated are the average number of patients in the first week and the percentage decrease compared to the pre-flood period in parentheses, as well as the respective average of the closure time is shown with the range in parentheses

Of all participating practices, 76.3% (*n* = 74) needed to close temporarily or permanently due to the flood (Fig. 2). In this group, the average number of the reported disruptions and damages was 7.7 compared to 4.1 for the 23 practices that remained open. In addition to the damages reported above and private affectedness, reasons given for closure included ‘time needed for tidying and cleaning’ (51.4%, *n* = 36), ‘time for necessary repairs’ (44.3%, *n* = 31), ‘lack of local accessibility’ (38.6%, *n* = 27) and ‘shortage of staff’ (5.7%, *n* = 4).

Since general practitioners not only represent the largest group in our survey but, as family doctors, also play an important role in the basic provision of outpatient care, we analyzed the practice closure situation of this group separately (Fig. 2). Of the 24 general practitioners, 66.7% (n = 16) reported that closure was necessary.

In general, the average duration of closure until reopening of the original practice was 27.7 days up to a maximum of 180 days (*n* = 56), with patient care partially compensated by the use of alternative premises after an average of 19.9 days up to a maximum of 90 days (*n* = 28) (Fig. 2). Specific designation of substitute practices for patient care during closure were indicated by 50.7% (*n* = 35). In 28 cases (80%) these were the usual substitute practices, e.g., for during holidays.

Of the 74 doctors who were unable to maintain patient care in their own practices, 56.8% (*n* = 42) saw a need for alternative premises and 43.2% (*n* = 32) stated that this was not necessary (e.g., due to a short period of closure) (Fig. 2). Regarding classification of alternative premises, 31.3% (*n* = 10) stated that they worked in a colleague's practice by themselves, 25.0% (*n* = 8) worked together with colleagues in their practice and 18.8% (*n* = 6) took care of their patients in public rooms not previously used for medical purposes. Other alternative premises included private rooms (6.3%, *n* = 2), German red cross mobile medical practices (6.3%, *n* = 2) and others (12.5%, *n* = 4) such as emergency practices or office containers. In most cases, the alternative premises were organized by the affected doctors themselves (46.2%, *n* = 12) or colleagues approached them with the offer (26.9%, *n* = 7).

Before the flood, the participating doctors saw an average of 206.5 patients per week (Fig. 2). Those offering home visits visited an average of 11.8 patients per week (*n* = 37). In the first week in the alternative premises, the number of patients decreased by 50.3% to an average of 66.2 patients per week. When resuming work in the original practice, whether immediately after the flood or after reopening, the doctors treated an average of 143.2 patients (*n* = 65) (decrease: -18.3%) in the first week. Those practices which remained open after the flood took care of 174.2 patients in the first week, a decrease of 13.4% (Fig. 2). In the group of those who had to close without the opportunity to work in alternative premises, there was a decrease of 20.9% in the first week after reopening if they stated alternative premises as necessary.

In the first month after the flood, 45.8% (*n* = 44) of the surveyed doctors treated fewer patients per week than before and 15.6% (*n* = 15) reported treating no patients at all during that period. 63.5% (*n* = 61) stated that there were substitution patients among the patients treated in the first month. Compared to previous substitution situations, the number of substitution patients was reported to be higher in 73.3% (*n* = 44) of the cases. Of those 47 who normally carry out home visits, 53.2% (*n* = 25) stated making fewer or no home visits in the month after the flood. In the group of general practitioners, who represent the majority of doctors performing home visits in general (51.1%, n = 24), only 2 (8.3%) reported an increased number during this period. 29 (70.7%) of all 41 doctors who made home visits after the flood reported that they had been limited in doing so by the flood and its consequences. Thereby, the local accessibility of the patients’ residences was seen as a problem by 89.3% (*n* = 25), the new accommodation of a part of the patients by 75.0% (*n* = 21), the lack of communication possibilities by 71.4% (*n* = 20) and 25.0% (*n* = 7) reported a risk due to building damage.

The local accessibility of the original practice before the flood was queried via agreement with various positive statements (Table 2). These were rated as rather or completely true in 90.7% of the cases for the situation before the flood. Evaluating the accessibility of the alternative premises after the flood, items such as ‘fully functional public transport’, ‘unimpeded approach by car’ and ‘barrier free access’ received particularly low agreement rates. For example, compared to the overall assessment of the local accessibility of the original practice before the flood, a decrease from an average of 90.8% agreement to 59.3% was recorded within the ‘alternative premises’-group. The statements regarding the local accessibility of the original practices after the flood were rated as rather or completely true in 51.1% of the cases overall, with 43.0% in the ‘closed without alternative premises’-group, 56.1% in the ‘remained open’-group and 68.8% in the group that reopened their original practices after working in alternative premises. Agreement was particularly low on the items ‘unimpeded approach by car’ and ‘fully functional public transport’. Compared to before the flood, the decrease was highest for the item "barrier-free access" (from 81.4% to 45.0%).

**Table 2:** Comparison of the local accessibility assessment

local accessibility	original practices before the flood				alternative premises (*n* = 28)	original practices after the flood			
percentage values of the answers "rather true" or "completely true" (%)^a^	all (*n* = 97)	closed		remained open (*n* = 23)		all (*n* = 80)	closed		Remained open (*n* = 22)
		alternative premises (*n* = 30)	NO alternative premise (*n* = 44)				Alternative premises (*n* = 14)	NO alternative premises (*n* = 44)	
**barrier-free access**	81.4	76.7	84.1	82.6	53.6	45.0	42.9	38.6	59.1
**walking access**	89.7	96.7	84.1	91.3	67.9	77.5	92.9	65.9	90.9
**access to public transport**	96.9	96.7	97.7	95.7	57.1	63.8	92.9	54.6	63.6
**fully functional public transport**	⏤	⏤	⏤	⏤	29.6	34.6 (*n* = 78)	61.5 (*n* = 13)	25.6 (*n* = 43)	36.4
**unimpeded approach by car**	⏤	⏤	⏤	⏤	44.4	27.5	50.0	18.2	31.8
**parking facilities**	94.9	93.3	95.5	95.7	78.6	57.5	71.4	54.6	54.6
**⌀ rather or completely true**	90.7	90.8	90.3	91.3	59.3	51.1	68.8	43.0	56.1

^a^ Percentage values of the answers "rather true" or "completely true" to the respective **positive** statements for the listed items in different subgroups. The number *(n)* of responses evaluated is given in parentheses in case they differ from the group size indicated in the heading

Patient care in the first week in the alternative premises was mainly impaired by ‘limiting phone failure’ (72.4%, *n* = 21), ‘more referrals’ (67.9%, *n* = 19), ‘longer appointment waiting times’ (64.3%, *n* = 18) and a ‘relevant lack of vaccines and drugs’ (64.0%, *n* = 16) (Table 3). Statements, such as ‘too few staff to provide optimal patient care’ (10.7%, *n* = 3) or ‘more patients than in a week before the flood’ (3.5%, *n* = 1) were reported less often. Doctors who worked in practices of colleagues (together or alone, *n* = 17) reported a more positive impression of patient care possibilities in this type of alternative premises, showing a significantly lower rate of agreement (p = 0.03) with the negative statements (38.2%) than doctors in other types of alternative premises (55.6%).

**Table 3:**
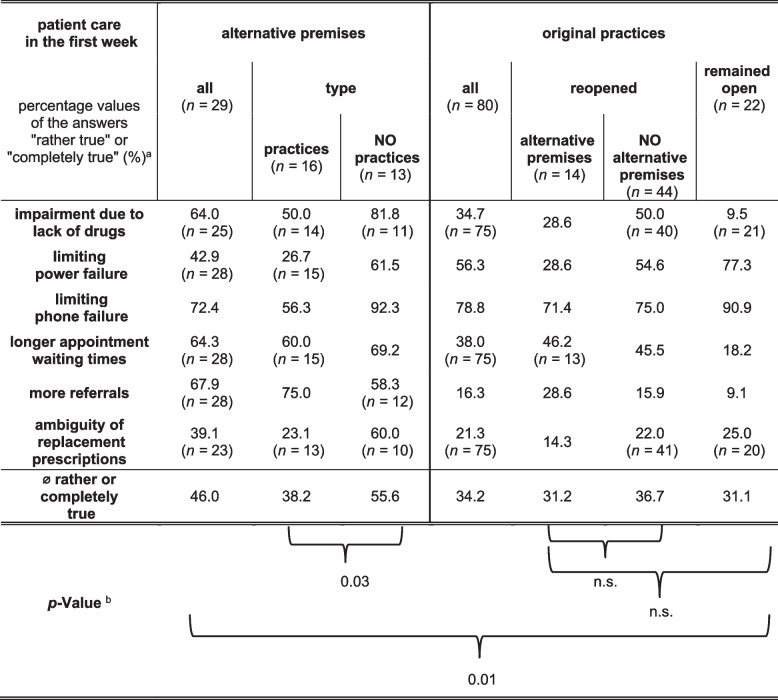
Comparison of the patient care assessment

^a^ Percentage values of the answers "rather true" or "completely true" to the respective **negative** statements for the listed items in different subgroups. The number (*n*) of responses evaluated is given in parentheses in case they differ from the group size indicated in the heading

^b^ *P*-Values are given for the comparison of the average agreement rate between different subgroups (n.s. = not significant)

In addition, the assessment of patient care was collected for the first week back in the original practice (Table 3). The doctors whose practices remained open (*n* = 22) and who thus directly attended patient care in the first week after the flood reported that telephone (90.9%, *n* = 20) and power failure (77.3%, *n* = 17) predominantly limited patient care. Further, there were ambiguities in issuing replacement prescriptions for lost drugs (25.0%, *n* = 5). In the group of those who had to close and could not use alternative premises (*n* = 44), telephone and power failure were also the leading limitations after reopening, but with lower agreement rates (75.0%, *n* = 33 and 54.6%, *n* = 24). Moreover, limitations due to ‘lacking vaccines, drugs, etc.’ (50.0%, *n* = 20) and ‘longer appointment waiting times’ (45.5%, *n* = 20) were frequently reported by this group.

To assess the assurance of outpatient care in the locality after the flood in general, the German school grading system (1 = very good to 6 = unsatisfactory) was used. The results revealed a score of 3.7 for the situation in the first week, 3.0 in the first month, and 1.8 at the time of the survey, approximately six months after the flood. The main problems reported were ‘less opened practices’ (91.7%, *n* = 88), the ‘limited local accessibility’ (86.6%, *n* = 84) and the ‘limited reachability by phone or email’ (84.4%, *n* = 81). Only 45.7% (*n* = 42) stated an ‘increased medical demand’.

Particularly disadvantaged in terms of access to health care after the flood were patient groups such as elderly/geriatric patients (65.4%, *n* = 51) and patients with disabilities (55.1%, *n* = 43), according to the participating doctors. Reduced mobility (81.3%, *n* = 61) and limitations in telephone communication (62.7%, *n* = 47) were stated as predominant reasons.

**Fig. 3:**
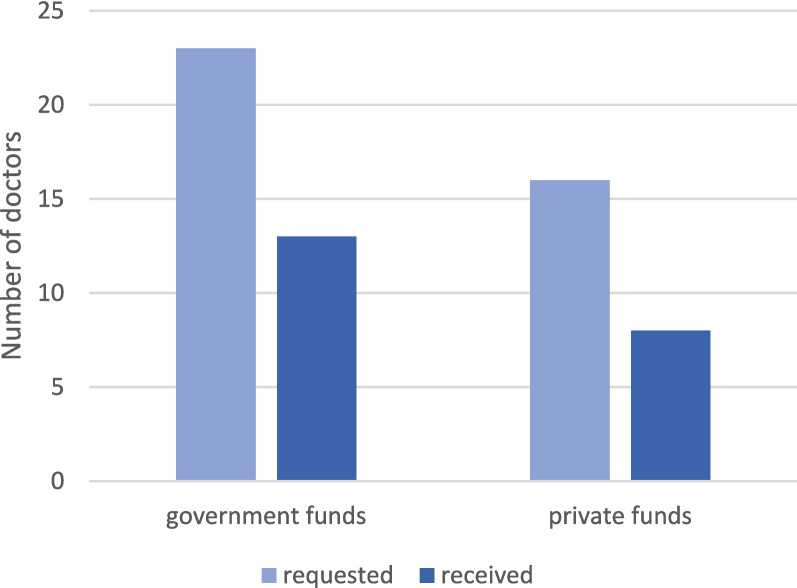
Number of applications for financial support and funds received

With regard to the offered measures and financial support to ensure outpatient care, participating doctors were asked which they knew about and which they applied for and received or used. On average, participants were aware of 73.3% of the different types of support available. The most frequent was the knowledge about financial support in form of emergency aid and reconstruction aid from the federal and state governments (92.3%, *n* = 84). Of the 25.3% (*n* = 23) who applied, 56.5% (n = 13) had received money at the time of the survey (Fig. 3). The option of financial support from private funds (e.g., donations collected by the KVNO) was known to 76.4% (*n* = 68), applied for by 18.0% (*n* = 16) and received by 9.0% (*n* = 8).

The approximately 20 free comments on the offered support in general and by the KVNO in particular cannot be analyzed statistically but apart from some notes such as *“[I] received a lot of information *via* the KV[NO] and tax consultant”* and *“The KVNO was unbureaucratic […]”* there was predominantly discontent about the support as well as the procedure (*“Employees of the KV[NO] and medical association [were] poorly informed”, “The KV[NO] has done almost nothing for us!”, “Help in financial form was only provided by practice insurance, authorities *etc*. were completely overwhelmed”, “The support provided by the KV[NO] went well at the beginning, but in the further course miserable.”, “I cannot say that the KV[NO] was helpful. Bureaucratic hurdles are high.”, “Due to the lack of internet / telephone connection for 11 weeks, I did not receive any information in the practice. Mail / written-postal would have been nice!”*).

## Discussion

To the best of our knowledge, this is the first study to examine the situation of outpatient care in the North Rhine region after the severe flood in July 2021. Going beyond most previous studies of post-natural disaster situations, we focused on the consequences on outpatient care rather than on direct health effects.

Regarding the methodological approach of our study, several aspects should be critically discussed. The media coverage of the flood event during the literature review may have biased the attention and perception of the researchers. As a consequence, it is possible that, for example, not all potential damages and thus alternative options for list selection have been fully considered. We aimed to overcome this by adding the option ‘other’ to all lists, with the possibility of more detailed specialization through free text comments. Furthermore, quality of health care is a complex construct defined by various indicators [28]. Thus, we did not presume to assess the actual quality of patient care in the post-flood period but grouped some indicators under the heading of "possibilities of outpatient care". In the context of a possible selection bias, the question arises whether there was a unifying factor between the practices that did not participate in the survey. It is conceivable that severely affected practices could not find the time to participate still dealing with the aftermath of the flood or that very slightly affected practices saw no need to participate. On the other hand, the inclusion of only affected practices and the presence of negative emotions as a potential unifying incentive to participate also contain the possibility of a selection bias. Moreover, the self-reporting nature of the questionnaire, together with the desire to raise awareness, may have caused a response bias.

The most interesting finding of our study was that the number of patients per week decreased sharply in all groups, with a maximum reduction in the group of doctors using alternative premises. Based on this data alone, it is not possible to determine whether the decline is due to a lower demand or limited ability to take care for the same number of patients as before the flood. Related research on a similar situation after a flood in the Midwest of the USA found that the ability to provide primary health care services decreased while medical demand, and therefore primary health care visits, increased [29]. However, when asked directly about the medical demand, more than half of the doctors participating in our study perceived no increase in the month following the disaster. This raises the question of possible explanations for why there was no clear increase in medical demand in the situation examined. Perhaps first responders, such as those from the German Red Cross, and hospital emergency rooms provided primary care for injuries sustained during the flood itself and during the cleanup afterwards, so fewer patients consulted established doctors for this purpose. Patients probably cancelled appointments for routine and preventive examinations, as was also observed despite an overall increase in medical demand in the above-mentioned study [29], possibly because these seemed to be of secondary importance to them at the time. The impact of that gap in medical care on chronical diseases in the affected region remains to be seen, but studies about cardiovascular diseases after natural disasters like hurricane Katrina at the Gulf coast of Mississippi and southeast Louisiana in 2005 suggest an increase of chronical diseases may be likely [30, 31].

Regarding the ability of providing outpatient care, the affectedness of health care workers with their private households and resulting staff shortages were rarely given as reason for closure and were only reported as minor limitations in our survey. An excerpt from a newspaper article interviewing a doctor whose private household was affected offers a possible explanation for why health care workers showed up for work in large part anyway. *"He tirelessly advocated for others–and to some extent, it's self-help. ‘There's a bit of normalcy in the practice; at home I no longer have a living room, a kitchen,'* [the interviewed doctor] […] *says.“* [20]. The results of other studies not only indicate that staff shortages can often lead to problems in patient care [32, 33] but have identified the workforce as the most important factor for a functioning public health service after natural disasters [34]. Accordingly, the results of our survey on this point are to be considered positive for securing outpatient care directly after the event in 2021.

Alongside the consideration of a temporary workforce reduction, permanent changes must also be taken into account. Even though only one doctor participating in our study indicated that there would be no reopening of the original practice, it can be suspected that there are more practices that will remain closed because the owner is close to retirement, rebuilding would take too long, or would not be financially worthwhile [20]. This group might be underrepresented in our study, *e.g*., for reasons of inaccessibility via practice contacts six months after the event or because they did not feel addressed by the questionnaire, since a large part of the questions is about patient care after the flood and thus could not be answered by them. Nevertheless, they should be considered when evaluating the loss of providers in the outpatient care setting.

Moreover, it is important to view all results of our study and comparisons with previous research in the context of the given circumstances that define the flood vulnerability of the population [35]. For the key indicators for public health and health system performance, Germany mostly meets or even exceeds the average, as stated in the 2021 Organisation for Economic Cooperation and Development (OECD) report ‘Health at a glance’ [36]. In contrast, many studies on health care after natural disasters were conducted in low to middle-income countries [35, 37] or in places where the health care system and access to it were already of limited quality for at least part of the population before the event [29]. Therefore, the decline in access to health care and the limitations that occurred in the period after the 2021 flood identified in our study should be viewed in relative terms. Also, the climate zone and prevailing hygiene standards are of great interest in assessing outcomes. Presumably, the high standard of hygiene, the absence of disease vectors and early instruction of the population in the handling of contaminated water ensured that there was no increase in flood-associated infectious diseases after the flood in 2021 [38–40]. Conversely, countries with different climate conditions and hygiene standards often experience an increase in diseases like malaria, typhus and cholera following disasters [35, 39, 41]. These circumstances could also be a possible explanation for the comparatively low medical demand found in our study.

In addition to considering the large-scale circumstances of the country, it is worthwhile to visualize the local conditions evoked by the flood via recalling the pictures from the affected areas. With trees lying in the streets, cars scattered in the way, missing bridges, roads impassable or completely washed away [10] and several hundred thousand tons of bulky waste [14], the explanation of the results for the local accessibility assessment of the practices is straightforward. Accordingly, the analysis revealed that even the practices that remained open or reopened were difficult to reach. The power outages that occurred due to damages and safety shutdowns to reduce the risk of water-related power accidents [10, 19] resulted, among other things, in the failure of elevators which most likely explains the leading decline in agreement with the statement "existing barrier-free access" after the flood. This is consistent with the participants’ indication that different groups, such as elderly / geriatric patients and patients with disabilities faced greater limitations in accessing health care, predominantly due to their limited mobility. Also, previous research has found that the elderly population was among the most vulnerable regarding access to primary health care due to lower mobility and adaptability as well as changes and loss of social infrastructure [29, 32].

In the post-disaster period, the above-mentioned limitations in local accessibility led among other things to the relocation of practices. Analysis of the possibilities of outpatient care in these alternative premises revealed differences between the several types. The evaluation of the situation in the first week indicates that the possibilities of patient care in practice premises were the best. After the disaster in July 2021, most of the affected doctors organized the alternative premises themselves or colleagues approached them with an offer. For the future, a distribution of doctors who lost their workplace by public authorities should be considered. A previously compiled list of practices that agree to host another doctor in the event of a disaster could provide as a basis. In the post-disaster situation, this could lead to an increase in the proportion of practices among the alternative premises with an anticipated improvement in the possibilities of patient care. In addition, there would also presumably be a reduction of the delay between closure of one's practice and the start of patient care in alternative premises. In this regard, Landeg et al. point out that *“[m]utual aid arrangements between health care providers need to reconsider the potential geographical scale of an event” *[33].

Another possible future implementation refers to the post-disaster communication with the affected doctors. In conversations during the questionnaire distribution and analysis of the free comments, the importance of the subject ‘offered measures and financial support’ for severely affected doctors became apparent. Doctors were already exhausted from coping with the corona pandemic and surprised by the refusal of funding after they lost everything and were still working in alternative premises at the time of the survey. Dissatisfaction and, to some extent, despair became apparent and can be seen as a kind of internal incentive to participate in our study and thus as one of the reasons for the comparatively high response rate. Moreover, the surveyed doctors drew our attention to the fact that information about possible measures was partly distributed by e-mail and therefore difficult to receive due to flood-related problems such as power failure. For future situations, a suggestion they provided was to use communication channels not being restricted by the aftermath of the disaster.

Even though communication via digital technologies failed in July 2021, digitalization is often seen as one of the ways to make the healthcare system more resilient to future events [42, 43]. Since it is certain that extreme weather events, such as extreme precipitation, will occur more frequently in the future [44], there is a need to increase natural disaster preparedness especially of critical infrastructure, such as the healthcare system. In 2010, for example, the WHO published a guidance document to review and improve the existing preparedness of hospitals and medical facilities [45]. Other tools for assessing the current situation include the U.S. National Health Security Preparedness Index, which in 2019 found a score of 6.7 out of 10 for overall patient care capabilities during and following a large-scale public threat and a score of 4.9 out of 10 for quality patient care capabilities for the United States [46]. A WHO survey in 2021 revealed, for the situation worldwide, that 63% of 177 countries reported a high to very high implementation status for health emergency management [47]. The level of natural disaster preparedness for outpatient care in particular is currently understudied. Nevertheless, the data from our study illustrate the need for further improvement in this area. As digital health technology (DHT) becomes increasingly important during natural disasters, its potential to improve outpatient care should be evaluated. In previous natural disasters in the United States or Australia, DHT was used to sustain ambulatory care through video consultations, provide medications via e-prescriptions, and share medical data through electronic medical records [42, 43]. However, the aftermath of the 2021 flood exposed DHT's vulnerability to disrupted connectivity, highlighting the need for DHT to become more climate resilient. Furthermore, the issue of unequal access to DHT needs to be addressed to ensure better outcomes for all individuals, including the vulnerable patient groups identified in our study.

In the context of learning from previous disasters to prepare the health care system for the future, it is necessary to overcome the phenomenon of "flood dementia". "Flood dementia" refers to the underestimation of a problem’s severity due to lack of personal involvement or confrontation, herein applied to flood consequences [48, 49]. Thus, experiences of people in one region have only a certain radius of effect and a certain duration of effect. An example of the short memory of flood consequences is the flood in Dresden in the year 2002. Already at that time, possible serious effects on inpatient health care in terms of hospital evacuations became apparent [50]. Subsequently, conclusions were drawn, such as that important hospital facilities, like electric power supply, should not be installed in flood-prone locations. Nevertheless, the 2021 flood, with hospital evacuations and flooded emergency power systems, showed that these experiences and analyses had not led to any change in the area now affected. Therefore, there is an urgent need to continue research on the drastic consequences of flood events and to draw attention to the necessary steps that need to be taken. This paper contributes to the search for cross regional solutions by quantifying the consequences of the 2021 flood and thus creating awareness.

## Conclusion

Following the 2021 flood, a high number of practices had to close due to flood consequences and the number of treated patients reduced sharply. Nevertheless, outpatient care in the region seemed to have been ensured. A possible explanation lies in the comparatively low post-disaster health vulnerability of the affected population, assuming good standards of public health performance and low risk of waterborne diseases in European high-income countries. Furthermore, we determined differences in the assessment of patient care depending on the type of premises and that affected doctors organized their relocation to alternative premises mainly privately.

Since extreme weather events will occur more frequently in the future, natural disaster preparedness of the health care system must be given great importance. The phenomenon of flood dementia needs to be overcome, so that assessments of previous disaster consequences are used to implement cross regional pre-emptive measures. Not locating health facilities in flood-prone areas, for example, could have avoided many consequences for outpatient care after the flood in 2021. Of course, not all damage caused by natural disasters can be forestalled. Therefore, our results indicate a number of possible measures to optimize post-disaster outpatient care. A distribution system could be established that allocates affected doctors to other practices in case of a natural disaster. Thereby, the potential geographical extent of a natural disaster should be taken into account. This might not only shorten the delay until restarting patient care, but also allow it to take place in practice premises with an anticipated improvement in the possibilities of patient care. Special consideration should also be given to post-disaster communication with affected physicians via unimpaired communication channels. In addition, DHT needs to be improved in terms of climate resilience and equality of access in order to realize its full potential for ensuring access to health care in the aftermath of natural disasters.

Although our study makes an initial contribution, further research would be of great interest. Establishing a distribution system for affected doctors in case of a natural disaster would require further research on the general feasibility and how different geographic extents of disasters could be considered. Given the greater flood extent in Rhineland-Palatinate with presumably more pronounced limitations in post-flood outpatient care, a survey in that region may also lead to interesting results regarding improvable processes.

## Data Availability

The datasets used and analyzed during the current study are available from the corresponding author on request.

## Abbreviations

ANOVA: Analysis of variance
DHT: Digital health technology
EDP: Electronic data processing
ENT: Ear, Nose and Throat
KVNO: Association of Statutory Health Insurance Physicians for North Rhine
KVs: Associations of Statutory Health Insurance Physicians
n.s.: Not significant
NRW: North Rhine-Westphalia
OECD: Organisation for Economic Co-operation and Development
OMS: Oral and maxillofacial surgery
RWTH: Rheinisch-Westfälische-Technische Hochschule Aachen
